# New Insights Into DAEC and EAEC Pathogenesis and Phylogeny

**DOI:** 10.3389/fcimb.2020.572951

**Published:** 2020-10-15

**Authors:** Mario Meza-Segura, Mussaret B. Zaidi, Arturo Vera-Ponce de León, Nadia Moran-Garcia, Esperanza Martinez-Romero, James P. Nataro, Teresa Estrada-Garcia

**Affiliations:** ^1^Molecular Biomedicine Department, Centro de Investigación y de Estudios Avanzados del Instituto Politécnico Nacional, Mexico City, Mexico; ^2^Infectious Diseases Research Unit, Hospital General O'Horan, Mérida, Mexico; ^3^Department of Epidemiology and Biostatistics, Michigan State University, Lansing, MI, United States; ^4^Centro de Ciencias Genómicas, Universidad Nacional Autónoma de México, Cuernavaca, Mexico; ^5^Department of Pediatrics, University of Virginia, Charlottesville, VI, United States

**Keywords:** DAEC, EAEC, genomes, phylogeny, virulence factors, DAEC pathogenesis

## Abstract

Diarrheagenic *E. coli* can be separated into six distinct pathotypes, with enteroaggregative (EAEC) and diffusely-adherent *E. coli* (DAEC) among the least characterized. To gain additional insights into these two pathotypes we performed whole genome sequencing of ten DAEC, nine EAEC strains, isolated from Mexican children with diarrhea, and one EAEC plus one commensal *E. coli* strains isolated from an adult with diarrhea and a healthy child, respectively. These genome sequences were compared to 85 *E. coli* genomes available in public databases. The EAEC and DAEC strains segregated into multiple different clades; however, six clades were heavily or exclusively comprised of EAEC and DAEC strains, suggesting a phylogenetic relationship between these two pathotypes. EAEC strains harbored the typical virulence factors under control of the activator AggR, but also several toxins, bacteriocins, and other virulence factors. DAEC strains harbored several iron-scavenging systems, toxins, adhesins, and complement resistance or Immune system evasion factors that suggest a pathogenic paradigm for this poorly understood pathotype. Several virulence factors for both EAEC and DAEC were associated with clinical presentations, not only suggesting the importance of these factors, but also potentially indicating opportunities for intervention. Our studies provide new insights into two distinct but related diarrheagenic organisms.

## Introduction

Worldwide, diarrheal disease remains a major cause of morbidity and is the eighth leading cause of mortality, among children under 5 years of age from less developed regions of the world (GBD 2016 Diarrhoeal Disease Collaborators, 2018). Diarrheagenic *Escherichia coli* pathotypes (DEP) are important etiological agents of acute and persistent diarrhea in children (Ochoa et al., 2009; Patzi-Vargas et al., 2015; Zhou et al., 2018), that can lead to death (Lanata et al., 2013; GBD 2016 Diarrhoeal Disease Collaborators, 2018), and cause traveler's diarrhea in adults (Paredes-Paredes et al., 2011; Jennings et al., 2017). Recent studies by our group have shown that DEP have become the main etiologic agents of diarrheal disease among children requiring hospitalization; DAEC and EAEC are the most prevalent pathotypes, surpassing *Salmonella* and *Shigella* (Patzi-Vargas et al., 2015).

DEP are classified into six well-described categories: shiga toxin producing *E. coli* (STEC), which encompasses the enterohemorrhagic *E. coli* group (EHEC); enterotoxigenic *E. coli* (ETEC); enteroaggregative *E. coli* (EAEC); enteroinvasive *E. coli* (EIEC); diffusely adherent *E. coli* (DAEC); and enteropathogenic *E. coli* (EPEC). The latter is subdivided into typical EPEC (tEPEC) and atypical EPEC (aEPEC) (Kaper et al., 2004). Overall, pathogenic *E. coli* use adhesins, sugars (capsular polysaccharides and lipopolysaccharides), toxins, invasion-promoting proteins, iron acquisition systems, complement resistance factors or immune evasion factors, to colonize, survive in, and injure their hosts (Kaper et al., 2004; Nataro, 2005; Meza-Segura and Estrada-Garcia, 2016). EAEC strains are characterized by their ability to form a stacked-brick or aggregative adherence (AA) pattern on HEp-2 cells, which is mediated by the aggregative adherence fimbriae (AAF) (Kaper et al., 2004). AAF transcription and that of at least other 44 EAEC chromosomal (aaiC) and plasmid-borne genes (the anti-aggregative secreted protein dispersin), are regulated by the Aggregative Adherence Regulator (AggR) (Nataro et al., 1994; Estrada-Garcia and Navarro-Garcia, 2012).

Due to the importance of AggR, EAEC isolates were subdivided into typical (tEAEC), i.e., *aag*R-positive, and atypical (aEAEC), i.e., *aag*R-negative, groups (Nataro, 2005). The recognition of tEAEC as strains carrying the *agg*R regulon was a major step in EAEC molecular identification, since it was shown that most EAEC strains previously characterized by the HEp-2 adherence assay harbored the *agg*R gene (Cerna et al., 2003). It is noteworthy that tEAEC, as well as other DEP, may also acquire other virulence factors (VF) by horizontal transfer and increase their virulence. In 2011, for example, a hybrid pathogenic EAEC-STEC O104:H4 that was responsible for a massive outbreak of bloody diarrhea in 16 countries, mostly harbored tEAEC (*agg*A, *agg*R, *set*1, *pic*, and *aap*) virulence genes and produced shiga toxin 2 (*stx*2) (World Health Organization, 2018).

Like EAEC, DAEC was classified based on its diffusely adherent (DA) pattern on HEp-2 cells, promoted by Afa, F1845, and Dr adhesins encoded by the *afa, daa*, and *dra* operons, respectively. Most of these adhesins have affinity for the human decay accelerating factor (hDAF; also known as CD55) or for carcinoembryonic antigen cell adhesion molecules (hCEACAMs), and both molecules are localized on the surface of intestinal epithelial cells (Servin, 2014; Meza-Segura and Estrada-Garcia, 2016). In contrast with EAEC, DAEC strains, aside from the adhesins, have only been associated with one other VF, the secreted autotransporter toxin (Sat) (Servin, 2014; Meza-Segura and Estrada-Garcia, 2016).

With the exception of tEPEC, aEPEC, STEC, and ETEC strains, the phylogenetic relationship among different DEP remains unclear (Lacher et al., 2007; Hazen et al., 2016; Montero et al., 2019; Rasko et al., 2019).

To date, not a single DAEC genome, and only a few different tEAEC genomes have been sequenced. Whole genome sequencing (WGS) of these pathotypes, in conjunction with previously reported *E. coli* genomes, will contribute to a greater understanding of the *E. coli* pan- and core-genomes. The pan-genome comprises the whole repertoire of genes identified in a bacterial species or group, while the core-genome includes the genes shared by all strains that encode for the proteins necessary for basic biological functions. *E. coli* species are considered to have an infinitely open pan-genome (Rouli et al., 2015), as it includes commensals and several pathogenic groups of strains capable of causing a wide variety of diseases, such as diarrhea, urinary tract infection, sepsis, and neonatal meningitis (Kaper et al., 2004). Therefore, *E. coli* phylogenetic analysis needs recognize *E. coli* pathogenic groups (Rasko et al., 2011; Rouli et al., 2015). WGS of DAEC and tEAEC may also help to identify new VF and potential novel pathogenetic mechanisms.

In this study, we sequenced the whole genome of ten DAEC and ten tEAEC strains identified as the sole pathogens isolated from the stools of patients with diarrhea, as well as one commensal *E. coli* strain isolated from a healthy child. These genomes were analyzed for their phylogenetic association with other *E. coli* strains and for the presence of genes encoding for VF. The results revealed that DAEC and tEAEC are phylogenetically related. However, strains of the different pathotypes harbor genes encoding for different sets of VF. DAEC carry more genes encoding for iron acquisition factors, while tEAEC harbor genes encoding toxins and bacteriocins. These findings will help to understand the molecular mechanisms that underlie the ability of DAEC and tEAEC to cause disease and will facilitate the identification of virulence gene profiles associated with more virulent strains. Moreover, this study will give insight into the origin and diversification of DAEC and tEAEC in reference to other DEP and ExPEC genomes.

## Materials and Methods

### Study Population

A 4-year study was conducted from March 2010 to July 2014 on 1,043 children < 10 years of age who sought medical care for acute community-acquired diarrhea at the Hospital O'Horan in Merida, Yucatan. The clinical severity of each diarrheal episode was determined by the Ruuska-Vesikari scoring system (RVSS) (Ruuska and Vesikari, 1990). In addition, children who presented bloody diarrhea but no shock, were classified as moderate diarrhea, and those who presented hypovolemic shock, severe electrolyte imbalance and/or seizures were classified as severe. On admission, a trained nurse obtained demographic and clinical information for each child on a standardized questionnaire. Stools from each child were collected and screened for *Escherichia coli, Campylobacter* spp., *Salmonella* sp., *Shigella* spp., *Vibrio* spp., and rotavirus, as previously described (Patzi-Vargas et al., 2015).

### Ethics Statement

This study was approved by both the Hospital General O'Horan Ethics Committee and the CINVESTAV Committee of Bioethics for Human Research. Legal guardians were required to sign an informed consent form. All children received medical treatment according to the hospital protocols.

### Identification of Diarrheagenic *E. coli*

From each patient, five *E. coli* like strains were selected. All isolates were both biochemically confirmed as *E. coli* and screened by two multiplex PCR for DEP genes. The first multiplex PCR identifies the presence of distinctive loci defining ETEC, EPEC, STEC and EIEC (Lopez-Saucedo et al., 2003), while the second multiplex PCR specific genes for DAEC and tEAEC (modified from Patzi-Vargas et al., 2013), including two plasmid-borne (*aap* and *aat*A) and one chromosomal (*aai*C) EAEC genes (Supplementary Table 1).

### DAEC and EAEC Strains Included for the VF -Clinical Severity Study

Thirty-eight DAEC and 30 EAEC strains isolated as the sole pathogen from children with diarrhea were selected for the characterization of 29 VF-encoding genes by four different multiplex PCR; one previously described (Guzman-Hernandez et al., 2016) and three newly developed in this study. The full description of the VF identified by each PCR assay, primers and PCR conditions, are described in Supplementary Table 1.

### DAEC and tEAEC Strains Included for WGS

For WGS, ten DAEC and nine tEAEC isolates were selected from those children with the most severe diarrhea who were not malnourished (Table 1). A tEAEC strain isolated as the only enteric pathogen from a 56-year-old woman with acute diarrhea, and a commensal *E. coli* strain from a healthy 2-year-old girl, both from Mexico City, were included for comparison.

**Table 1:** Patient's clinical profile and strains genome characteristics.

**Group**	**Patient**	**Gender**	**Age (years)**	**DWD**	**DWV**	**Complications**	**Clinical outcome**	**Strain**
**DAEC**	CA0022	M	2.3	3	1	None	Moderate	MEX-1
	CA0030	M	4.8	5	3	None	Severe	MEX-2
	CA0063	F	2.4	4	3	None	Moderate	MEX-3
	CA0170	M	0.1	4	1	S	Severe	MEX-4
	CA0209	F	1.5	7	2	HS, S	Severe	MEX-5
	CA0273	F	1.1	8	1	None	Moderate	MEX-6
	CA0437	M	1.8	6	2	BIS, HN	Severe	MEX-7
	CA0472	M	1.0	8	1	None	Severe	MEX-8
	CA0582	M	2.7	5	2	HN, HK	Severe	MEX-9
	CA1035	M	0.9	5	1	None	Moderate	MEX-10
**tEAEC**	CA0008	F	0.9	3	2	None	Moderate	MEX-11
	CA0036	F	2.8	2	1	BIS	Severe	MEX-12
	CA0041	M	1.3	5	3	BIS	Severe	MEX-13
	CA0155	M	1.5	4	1	None	Mild	MEX-14
	CA0345	F	0.6	6	1	None	Moderate	MEX-15
	CA0577	F	1.4	6	2	HN, HK	Severe	MEX-16
	CA0603	M	1.3	8	4	HN, HK	Severe	MEX-17
	CA0714	M	1.1	7	1	None	Moderate	MEX-18
	CA1036	F	1.2	9	1	HN, S	Severe	MEX-19
	TE-001	F	54.0	3	1	None	Moderate	MEX-20
**Commensal**	SE-001	F	2.0	–	–	–	–	MEX-21

*DWD, Days with diarrhea; DWV, Days with vomit; HS, Hypovolemic shock; S, seizures; BIS, Blood in stool; HN, Hyponatremia; HK, hypokalemia*.

Genomic DNA was extracted by a phenol/chloroform method (Neumann et al., 1992). DNA libraries were prepared using the illumine TrueSeq DNA nano sample preparation kit and sequenced on the Illumina MiSeq platform at CINVESTAV-Langebio to generate tagged paired-end reads of 250 bases in length. Quality control, trimming and filtering of raw sequencing data was performed using Trim Galore v0.4.1^1^. Genomes were assembled with SPAdes v3.9.0 (Nurk et al., 2013; Prjibelski et al., 2014; Vasilinetc et al., 2015), and the accuracy of the assemblies was evaluated with REAPR 1.0.16 (Hunt et al., 2013). Pre-assembled contigs were scaffolded using SSPACE_Standard_v3.0.pl (Boetzer et al., 2011) and GapFiller_v1-10 (Boetzer and Pirovano, 2012).

### Phylogenomic Analysis

Phylogenetic trees were constructed using a total of 107 genomes, including the 21 genomes sequenced in this study and 79 publicly available genomes for pathogenic *E. coli*, six commensal *E. coli* and one *Escherichia fergusonii* (Supplementary Table 2). Open reading frames (ORFs) of all genomes were predicted by GeneMark (Besemer and Borodovsky, 2005) and a Maximum Parsimony pan-genome phylogenetic tree was constructed with GetHomologues (Contreras-Moreira and Vinuesa, 2013), based on the translated protein sequences of each genome, using a combination of the orthoMCL and COGS as clustering algorithms. Single-copy genes of the *E. coli* core-genome were retrieved with a custom Bash/Perl script deposited in Github^2^ MAFFT 7.3.10 was used to align all sequences (Katoh and Standley, 2013). After alignment all sequences were concatenated using the multigenomes2blocks pipeline (Vera-Ponce de Leon et al., 2020) and the GTR+F+R10 nucleotide substitution model was obtained by ModelFinder (Kalyaanamoorthy et al., 2017). The maximum-likelihood (ML) phylogenetic tree was then calculated by IQtree v1.6.12 (Minh et al., 2020) with 1,000 Bootstrap replicates for internal branch support. Population structure was calculated using a Bayesian analysis approach (BAPS) by RhierBAPS package (Tonkin-Hill et al., 2018) in R 3.6.3. For this, two depth levels and a maximum clustering size of 20 (default = number of isolates/5; 107/5 = 21.4) were specified. Seven different sequence clusters (SC) were identified, which were further divided into 24 lineages (Supplementary Figure 1). All scripts to replicate this experiment are deposited in Github^2^. Pan- and core-genome phylogenetic trees were visualized and edited using iTOL (Letunic and Bork, 2016).

### Identification of ORFs Encoding for Virulence Factors

For this study, we defined VF as molecules that enable a microorganism to establish itself on or within a host of a particular species and enhance its potential to cause disease. A commensal *E. coli* strain was defined as an isolate from the stool of subjects exhibiting no signs of diarrheal illness and that did not display any known molecular markers that are associated with DEP. The presence of genes encoding for bacterial VF was analyzed by a Protein-Protein BLAST (BLAST 2.2.28+), using the translated protein sequences of all predicted ORFs and the virulence factor database (VFDB) (Liu et al., 2019). VF were annotated when their coverage and identities were ≥ 80%, after BLAST searches. VF were included for the analysis only when all genes or operons involved in their production or secretion were identified (i.e., the Sit iron/manganese transport system was only included when the genes encoding for *sit*A, *sit*B, sitC, and *sit*D were present). Individual VF were grouped together based on their function, including adhesins, bacteriocins, toxins, iron acquisition systems (IAS), and complement resistance or immune system evasion factors (CR/ISEF). The relative frequency (RF) of these groups was calculated by adding together the individual frequency of each VF in that group and dividing the result by the total number of genomes.

### Statistical Analysis

All statistical analyses were performed using GraphPad PRISM® version 5.0 software. The incidence of individual VF was evaluated by Fisher's exact test (FET) and the RF of VF groups by Mann-Whitney *U-*Test (MWUT). *P* < 0.05 were considered statistically significant.

### Data Availability

The genome sequence assemblies generated in this study have been deposited in GenBank as part of the Bioproject PRJNA588567 under the accession numbers listed in Supplementary Table 2. The remaining data that support the findings of this study are available from the corresponding author upon request.

## Results and Discusion

### Pan-Genome and Core-Genome of *E. coli*, DAEC, and EAEC

The clinical characteristics of each subject and the general genomic features of their sequenced strains are summarized in Table 1 and Supplementary Table 3, respectively. Almost all children presented with moderate to severe diarrhea (95%) and were < 5 years of age (95%). *E. coli* genome sizes obtained ranged from 4.9 to 5.5 Mbp, similar to those reported for other DEP genomes (Lukjancenko et al., 2010). To our knowledge, these are the first DAEC genomes to be reported.

In order to establish the phylogenetic relationships among the 21 genomes described in this work, 85 previously sequenced *E. coli* genomes were used for comparison, including six tEAEC, one aEAEC and ten tEAEC-STEC O104:H4, forty-nine DEP, ten uropathogenic *E. coli* (UPEC), three neonatal meningitis-causing *E. coli* (NMEC), six commensal and MG1655 K12 genomes (Supplementary Table 2). *E. fergusonni* (ATCC® 35469™) (Farmer et al., 1985) was used as an outgroup to root the phylogenetic trees. The comparative genomic analysis revealed a pan-genome containing 25,726 genes, including 1,452 core-genes, of which only 1,144 were single copy. This pan-genome contained more genes than those previously described with a similar number of genomes analyzed, reported with 13,000–16,373 genes (Rasko et al., 2008; Lukjancenko et al., 2010; Kaas et al., 2012). The number of genes included in the core-genome, however, were comparable to previously published studies, in which an average number of 1,631 genes was reported (Rasko et al., 2008; Lukjancenko et al., 2010; Kaas et al., 2012).

### Pan- and Core-Genome Phylogenetic Clades Revealed DAEC and EAEC Relatedness

**Figure 1:**
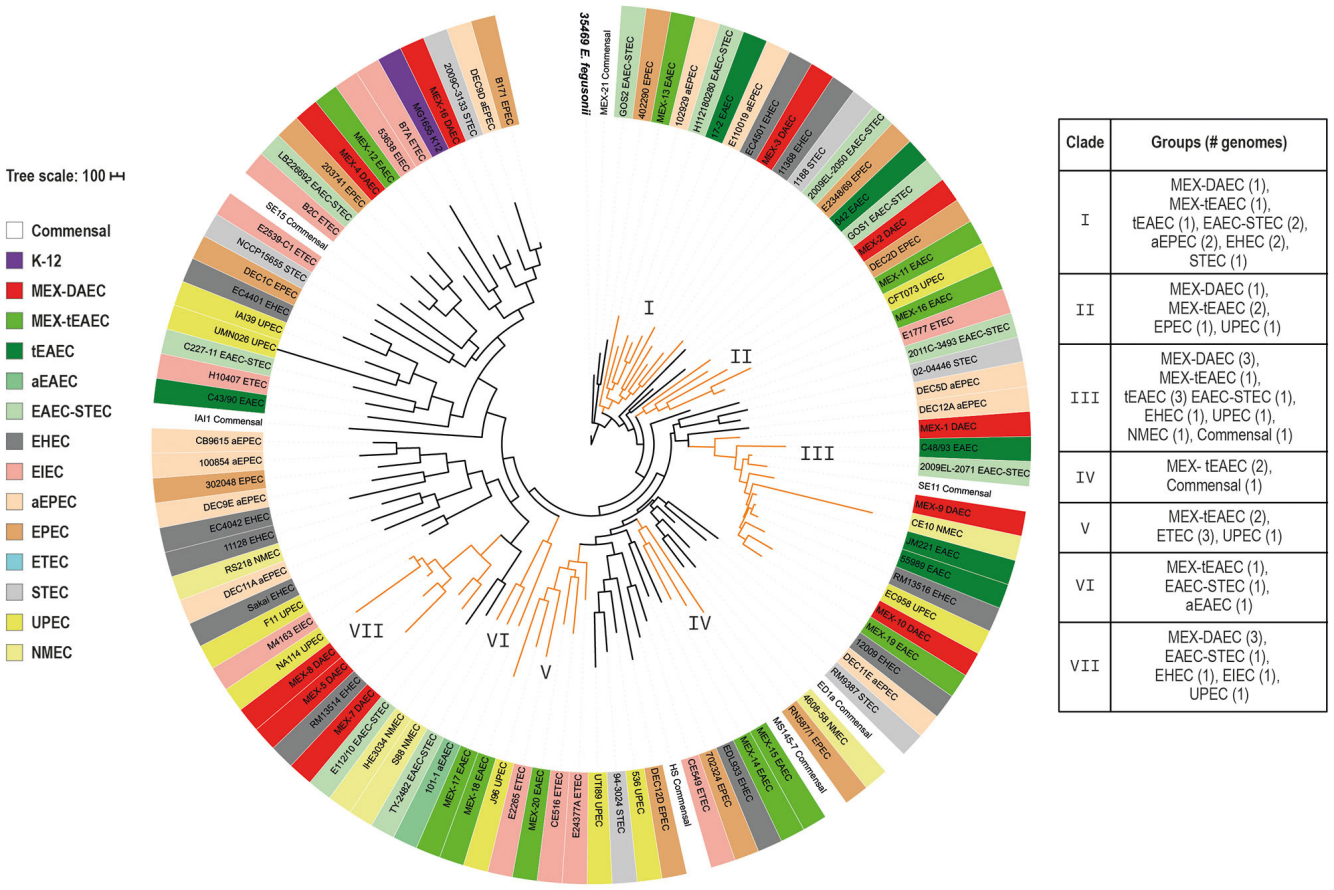
Pan-genome phylogenetic tree. The tree was built up based on the presence or absence of 25,726 genes comprised in the *E. coli* pan-genome. *E. fergusonii* 35,469 (in bold) was included to root the tree. Each *E. coli* group is indicated by a different color listed in the left box. Clades including more than one DAEC or tEAEC genomes are highlighted in orange and the roman numeral indicates the clade number. The scale bar represents the number of differences in the presence or absence of genes among all genomes.

For the pan-genome tree we selected those clades harboring more than one DAEC or tEAEC genomes (I–VII), each of which included some of the sequenced strains (Figure 1). Clade VI contained only one tEAEC strain sequenced in this study, as well as one hybrid EAEC-STEC and one aEAEC reference strain, revealing that these three EAEC groups share a common set of genes.

**Figure 2:**
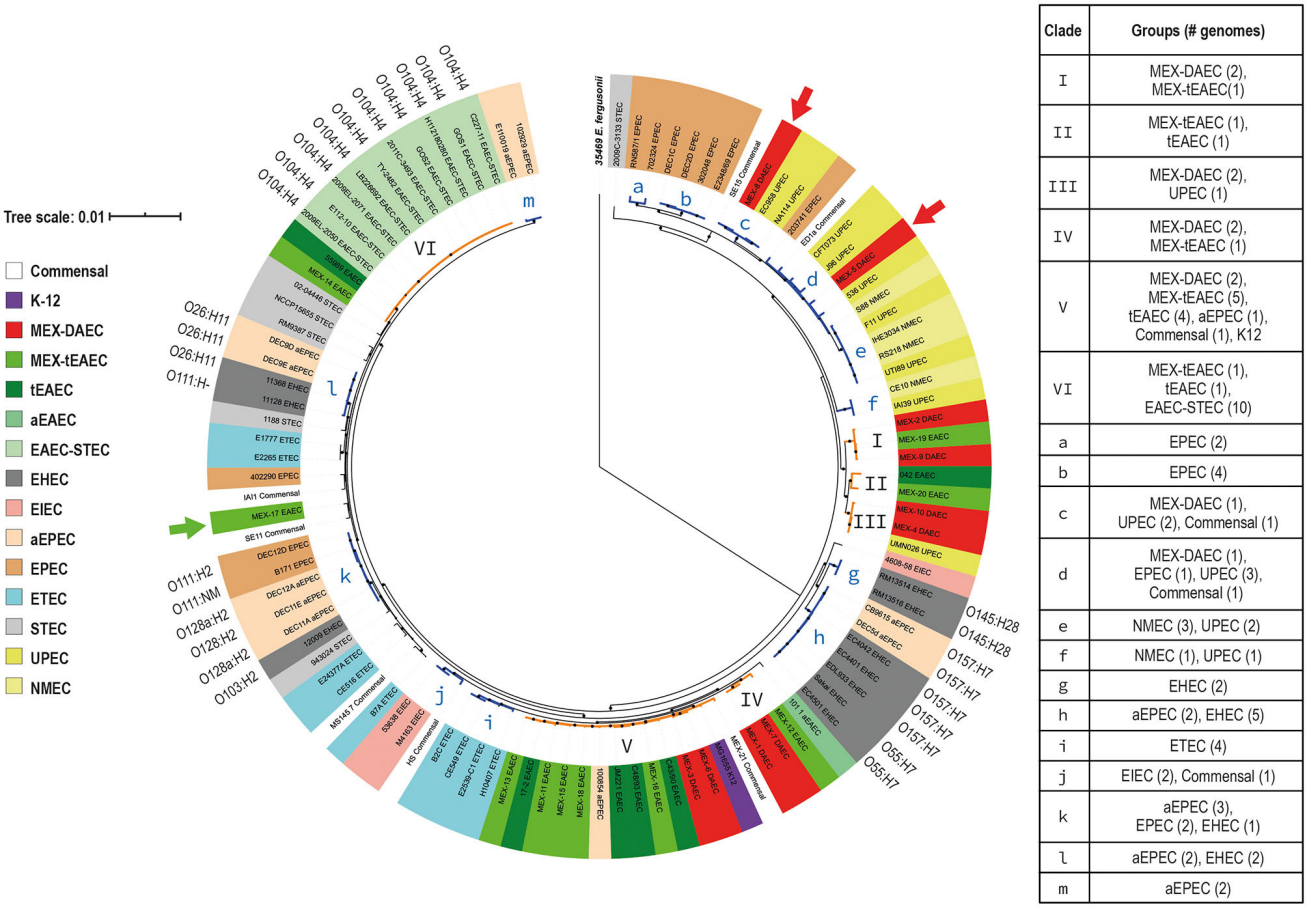
Core-genome (GTR + F + R10) phylogenetic tree. A maximum likelihood tree was constructed based on 1,144 single copy genes from the *E. coli* core-genome. *E. fergusonii* 35,469 (in bold) was used as an out-group. The tree was then calculated by IQtree v1.6.12 (Minh et al., 2020) with 1,000 Bootstrap replicates for internal branch support and values over 70% are indicated at each node with a black circle. Population structure was calculated using a Bayesian approach by RhierBAPS package (Tonkin-Hill et al., 2018) in R 3.6.3. *E. coli* groups are indicated by different color labels listed in the left chart. Clades including more than one DAEC or tEAEC genomes are highlighted in orange and the roman numerals in front of them indicate the clade number. Two DAEC (red arrows) and one tEAEC (green arrow) genomes were located outside these clades. Clades highlighted in blue (a–m) contain other pathogenic *E. coli* genomes that had important associations. The scale bar represents the number of nucleotide substitutions per site.

To analyze the evolutionary relationships among DEP we built a core-genome phylogenetic tree, and the population structure was analyzed using a Bayesian approach. All 107 genomes were grouped into seven sequence clusters (SC), which were further divided into 24 lineages (Supplementary Figure 1). Nineteen of these lineages were selected and illustrated in the core-genome tree as clades I-VI and a-m (Figure 2). The tree revealed six different phylogenetic origins for each DAEC and tEAEC pathotypes. Even though these two DEP were outnumbered by other *E. coli* genomes, clades I–VI mostly comprised DAEC or tEAEC strains. Clades I and IV exclusively contained DAEC and tEAEC genomes. Additionally, clade V, which mostly harbored these two pathotypes (two DAEC and nine tEAEC), also contained one aEPEC, one commensal and K12 strains. Altogether, this results strongly suggest that DAEC and tEAEC are phylogenetically related. Of note, nine of the 14 genomes (64.3%) included in clade V were isolated from Mexican children, suggesting a geographical cluster: two MEX-DAEC, five MEX-tEAEC, the commensal MEX-21 and the JM221 tEAEC reference strain, which was isolated from a Mexican child with diarrhea in the 1980s (Mathewson et al., 1987). Clade VI solely contained tEAEC and EAEC-STEC O104:H4 genomes, confirming that EAEC-STEC O104:H4 is more closely related to tEAEC than to EHEC 0157:H7 (Rasko et al., 2011).

The core-genome tree also revealed that DAEC clustered together with UPEC strains (clades III, c, and d). Clade III and c harbored exclusively DAEC and UPEC genomes, whereas clade d also included an aEPEC and a commensal strain. This was not unexpected, given that UPEC strains carrying *afa*/*daa*/*dra* operons have been identified among UTI patients (Servin, 2014).

Additionally, the core-genome tree revealed associations between other *E. coli* groups (clades a, b, e–m). Clades a and b encompassed typical EPEC strains that resemble previously described EPEC9 and EPEC1 phylogenomic lineages, respectively, whereas clade m belongs to the B1 phylogroup and comprises two aEPEC strains, including the prototype E110019 strain (Lacher et al., 2007; Hazen et al., 2016). Moreover, clade h supports the notion that EHEC O157:H7 evolved from O55:H7 aEPEC (Zhou et al., 2010). Clade k harbors EPEC, aEPEC and EHEC genomes, similar to EPEC2 phylogenomic lineage (Lacher et al., 2007; Hazen et al., 2016). Phylogenetic relatedness was also observed between aEPEC and EHEC strains belonging to the O26:H11 serotype (clade L); these strains undergo transient interconversions via loss and gain of Stx-encoding phages (Bielaszewska et al., 2007).

Of interest, four ETEC (40%) and two EIEC (66.6%) genomes formed clades i and j, respectively, suggesting a specific phylogenetic origin for each pathotype (Rasko et al., 2019). In addition, clades e and f, containing NMEC and UPEC strains, revealed that these ExPEC groups are phylogenetically related. The fact that commensal *E. coli* genomes were widely distributed and clustered with strains from different pathogenic groups, confirms the great diversity of *E. coli* genomes (Figure 2).

### Analysis of DAEC and tEAEC VF by Function Reveals Different and Novel Pathogenic Mechanisms

In order to identify new DAEC and tEAEC pathogenic mechanisms, virulence factors were grouped by function: adhesins, bacteriocins, toxins, iron acquisition factors (IAF), complement resistance or Immune system evasion factors (CR/ISEF), and bacterial secretion systems. With the exception of adhesins, all genes identified among these groups are mentioned in Table 2. In addition, the genes encoding for the following 14 adhesins were identified: antigen 43 (Agn43), calcium-binding antigen 43 homolog (Cah), curli, EaeH surface protein, *E. coli* common pilus (ECP), EHEC autotransporter encoding genes A (EhaA) and B (EhaB), *E. coli* YcbQ laminin-binding fimbriae (Elf/Ycb), pyelonephritis-associated pilus (Pap), Stg fimbria, toxigenic invasion loci A/haemagglutinin from *E. coli* K1 (Tia/Hek), type 1 fimbria (TIF), trimeric autotransporter adhesin UpaG, and autotransporter adhesin UpaH. Most of these adhesins have been implicated in adhesion to epithelial cells, autoaggregation and biofilm formation. The relative frequency (RF) of adhesins was similar among DAEC (RF 7.4), tEAEC (RF 7.5), and commensal (RF 7.0) *E. coli* strains, revealing that adherence is a key event for initiating *E. coli* commensal and pathogen colonization, and that its success relies on multiple adhesins that recognize receptors on the intestinal and bladder epithelium (Kaper et al., 2004; Flores-Mireles et al., 2015).

**Table 2:** Distribution of groups of virulence factors among DAEC, EAEC, and commensal *E. coli* genomes.

**Genome**	**Bacteriocins**		**CR/ISEF**		**IAF**		**Toxins**		**Secretion systems**		**Total**
	**#**	**VF**	**#**	**VF**	**#**	**VF**	**#**	**VF**	**#**	**VF**	**#**
MEX-1-DAEC	1	CcdAB	2	OmpA, TraT	4	Aer, Ent, Sit, Ybt	0	–	0	–	7
MEX-2-DAEC	0	–	4	Iss, KPS, OmpA, TraT	5	Aer, Ent, Chu, Sit, Ybt	3	HlyE, Sat, TieB	1	ETT2	13
MEX-3-DAEC	1	CcdAB	4	Iss, KPS, OmpA, TraT	4	Aer, Ent, Sit, Ybt	3	HlyE, Sat, TieB	0	–	12
MEX-4-DAEC	1	CcdAB	4	Iss, KPS, OmpA, TraT	5	Aer, Chu, Ent, Sit, Ybt	2	HlyE, Sat	1	ETT2	13
MEX-5-DAEC	1	CcdAB	5	Iss, KPS, OmpA, TcpC, TraT	4	Aer, Chu, Ent, Ybt	1	Vat	0	–	11
MEX-6-DAEC	1	CcdAB	4	Iss, KPS, OmpA, TraT	4	Aer, Ent, Sit, Ybt	3	HlyE, Sat, TieB	0	–	12
MEX-7-DAEC	1	CcdAB	3	Iss, OmpA, TraT	4	Aer, Ent, Sit, Ybt	0	0	0	–	8
MEX-8-DAEC	1	CcdAB	3	KPS, OmpA, TraT	6	Aer, Chu, Ent, Hbp, Sit, Ybt	3	EspC, Sat, TieB	0	–	13
MEX-9-DAEC	0	–	3	Iss, KPS, OmpA	3	Chu, Ent, Ybt	3	EAST1, EatA, HlyE	1	ETT2	10
MEX-10-DAEC	1	CcdAB	4	Iss, KPS, OmpA, TraT	4	Chu, Ent, Sit, Ybt	2	HlyE, Sat	1	ETT2	12
**RF**	0.80		3.60		4.30		2.00		0.40		11.10
MEX-11-tEAEC	1	CcdAB	4	Iss, KPS, OmpA, Pic	3	3: Aer, Ent, Ybt	2	2: HlyE, ShET1	1	Aai	11
MEX-12-tEAEC	1	MccH47	3	OmpA, Pic, TraT	2	EntA, Ybt	3	3: EAST1, HlyE, ShET1	1	Aai	10
MEX-13- tEAEC	2	CcdAB, MccH47	4	Iss, KPS, OmpA, Pic	3	Aer, Ent, Ybt	5	EAST1, HlyA, HlyE, Sat, ShET1	1	Aai	15
MEX-14-EAEC	2	CcdAB, MccH47	2	OmpA, Pic	3	Aer, Ent, Ybt	5	HlyA, Pet, SepA, ShET1, SigA	1	Aai	13
MEX-15-EAEC	2	CcdAB, MccH47	2	OmpA, Pic	2	Ent, Ybt	4	EAST1, HlyE, Sat, ShET1	1	Aai	11
MEX-16-EAEC	1	CcdAB	3	OmpA, Pic, TraT	1	Ent	2	2: HlyE, ShET1	1	Aai	8
MEX-17-EAEC	1	CcdAB	2	OmpA, Pic	3	Aer, Ent, Ybt	4	EAST1, Sat, SepA, ShET1	1	Aai	11
MEX-18-EAEC	1	CcdAB	2	Iss, OmpA	3	Ent, Sit, Ybt	1	HlyE	0	–	7
MEX-19-EAEC	1	CcdAB	3	KPS, OmpA, TraT	5	Aer, Chu, Ent, Sit, Ybt	3	HlyA, HlyE, Sat	1	ETT2	13
MEX-20-EAEC	0	–	5	Iss, KPS, OmpA, Pic, TraT	4	Chu, Ent, Sit, Ybt	4	EAST1, HlyE, SepA, ShET1	1	Aai	14
042-EAEC	2	CcdAB, MccH47	4	KPS, OmpA, Pic, TraT	4	Chu, Ent, Sit, Ybt	4	EAST1, HlyE, Pet, ShET1	2	Aai, ETT2	16
55989-EAEC	1	CcdAB	2	OmpA, Pic	3	Aer, Ent, Ybt	4	EAST1, Pet, ShET1, SigA	1	Aai	11
C43/90-EAEC	2	CcdAB, MccH47	3	OmpA, Pic, TraT	3	Aer, Ent, Ybt	4	EAST1, HlyE, Sat, ShET1	1	Aai	13
48/93-EAEC	2	CcdAB, MccH47	3	Iss, OmpA, Pic	3	Aer, Ent, Ybt	3	HlyE, Sat, ShET1	1	Aai	12
JM221-EAEC	1	CcdAB	3	Iss, OmpA, Pic	3	Aer, Ent, Ybt	3	HlyE, Sat, ShET1	1	Aai	11
17-2-EAEC	1	CcdAB	3	Iss, KPS, OmpA	4	Aer, Ent, Sit, Ybt	5	EAST1, HlyA, HlyE, Sat, ShET1	1	Aai	14
**RI**	1.31		3.00		3.06		3.50		1.00		11.88
MEX-21-Com	0	–	3	Iss, OmpA, TraT	3	Aer, Ent, Sit	1	HlyE	0	–	7
HS-Com	0	–	1	OmpA	1	Ent	0	–	0	–	2
SE11-Com	1	CcdAB	2	Iss, OmpA	1	Ent	0	–	0	–	4
SE15-Com	1	CcdAB	3	KPS, OmpA, TraT	4	Chu, Ent, Sit, Ybt	2	EspC, TieB	0	–	10
IAI1-Com	0	–	2	Iss, OmpA	1	Ent	0	–	0	–	3
ED1A-Com	1	CcdAB	2	KPS, OmpA	5	Aer, Chu, Ent, Sit, Ybt	2	Sig, TieB	0	–	10
MS145-7-Com	1	CcdAB	2	OmpA, TraT	1	Ent	0	–	0	–	4
**RI**	0.57		2.14		2.29		0.71		0.00		5.71

*CR/ISEF, Complement resistance/Immune system evasion factors; IAF, Iron acquisition factors; RI, Relative incidence; VF, Virulence factors; CcdAB, CcdA/CcdB type-II toxin-antitoxin system; MccH47, Microcin H47; Iss, Increased serum survival protein; KPS, group II Capsule; OmpA, Outer membrane protein A; Pic, Protein involved in colonization; TcpC, Tir domain containing protein; ChuA, E. coli hemin uptake system; Ybt, Yersiniabactin; Aer, Aerobactin; SitA, Sit iron/manganese transport system; EAST1, EAEC heat stable toxin 1; HlyA, α-Hemolysin; HlyE, Hemolysin E; Pet, Plasmid encoded toxin; Sat, Secreted autotransporter toxin; SepA, Secreted serine srotease A; ShET-1, Shigella enterotoxin 1; SigA, Shigella IgA-like protease homolog; Vat, Vacuolating autotransporter toxin; Aai, Aai-type VI secretion system; ETT2, E. coli Type III secretion system 2*.

Since the information on DAEC VF is scarce, it was interesting to find that DAEC harbored significantly more IAF than both tEAEC (4.30 vs. 3.06 *p* = 0.0023) and commensal *E. coli* (4.30 vs. 2.29 *p* = 0.0256), suggesting that IAF may play an important role in DAEC virulence (Table 2). Iron is essential for *E. coli* survival, facilitating numerous essential cellular activities, such as peroxide reduction, electron transport, and nucleotide biosynthesis (Gao et al., 2012). In other pathogenic *E. coli* groups, such as UPEC, IAS play an important role in virulence *in vivo* (Torres et al., 2001; Flores-Mireles et al., 2015). *Shigella* has also evolved multiple strategies for sequestering iron and other elements from the host (Wei and Murphy, 2016).

As shown in Table 2, tEAEC carried significantly more bacteriocins than DAEC (*p* = 0.0005) and commensal *E. coli* (*p* = 0.0167). tEAEC genomes were the only ones to harbor the Microcin H47 (43%) system. Microcin H47 was first found in the non-pathogenic, probiotic Nissle 1917 *E. coli* strain and is highly prevalent among ExPEC strains (Micenkova et al., 2014). It exhibits antibacterial activity against enteric pathogens such as Adherent Invasive *E. coli* (AIEC) and *S. enterica* serovar Typhimurium (Sassone-Corsi et al., 2016; Palmer et al., 2018). It is plausible that DAEC, and particularly tEAEC, use bacteriocins to eliminate competing bacteria in the gut, including other pathogenic species. Likewise, tEAEC had significantly more toxins (Figure 2) than DAEC (3.50 vs. 2.0, *p* = 0.007) and commensal *E. coli* (3.50 vs. 0.71, *p* = 0.0321), providing evidence that toxins play an important role in tEAEC virulence (Estrada-Garcia and Navarro-Garcia, 2012). DAEC had significantly more toxins than commensal strains (2.00 vs. 071, *P* = 0.0418), thus maybe toxins play a role in DAEC pathogenesis as well.

Two secretion systems were identified among the sequenced genomes: (1) the *E. coli* Type III secretion system 2 (ETT2), which was first discovered through the genome analysis of enterohemorrhagic *E. coli* O157:H7 (Ren et al., 2004), and (2) the tEAEC Aai type six secretion system (T6SS), which is regulated by AggR and its function is as yet unknown (Dudley et al., 2006).

ETT2 was more prevalent in DAEC (40%) than in tEAEC (12.5%) and was absent in all commensal strains (Table 2). With exception of MEX-20 tEAEC genome, the whole ETT2 gene cluster was identified in all strains of clades I, II, III, g and h. It has been reported that ETT2 participates in the invasion of endothelial cells by NMEC strain EC10 (Yao et al., 2009) and may have functional importance in infection among human pathogenic *E. coli* strains isolated from blood samples (Fox et al., 2020). Aai-T6SS was only identified among tEAEC genomes (*P* < 0.0001). T6SS was initially associated with bacterial virulence in eukaryotic host cells, but currently the main role of T6SS is thought to involve bacterial competition via killing of neighboring bacteria by the injection of antibacterial proteins directly into their periplasm after cell-cell contact (Navarro-Garcia et al., 2019).

CR/ISEF were significantly more prevalent in DAEC (RF 3.60 vs. 2.14, *P* = 0.0043) and tEAEC (RF 3.00 vs. 2.14, *P* = 0.0008) than among commensal strains, but there was no difference between these two pathotypes. CR/ISEF may play an essential role in DAEC pathogenesis, since the initial binding of Afa/F1845/Dr adhesins to hDAF could potentially result in a decreased inactivation of the complement cascade, thus production of CR/ISEF could protect DAEC from the action of complement (Duan and Mukherjee, 2016; Meza-Segura and Estrada-Garcia, 2016).

### Novel and Common VF Genes Found Among DAEC and tEAEC Strains

Because very little is known about DAEC VF this section highlights the new VF genes identified for this pathotype. We also describe common tEAEC and commensal VF genes (Table 3). When compared to tEAEC, Cah was highly prevalent among DAEC genomes (90% vs. 37.5%, *p* = 0.0143), and was absent from commensal bacteria (Table 3). *cah* has been described to be widespread among STEC and has only been identified in one commensal *E. coli* strain (Torres et al., 2002; Montero et al., 2014; Carter et al., 2018). Cah shares high sequence similarity with AIDA-1, an adhesin that mediates DAEC diffuse adherence pattern to HeLa cells, and with Antigen 43. In *E. coli* O157:H7, Cah is known to be involved in autoaggregation and biofilm formation (Carter et al., 2018). The gene encoding for the enterotoxin TieB (*sen*B) was also significantly more prevalent in DAEC, since it was absent in tEAEC genomes (*p* = 0.0140, Table 3); there was no significant difference between DAEC and commensal *E. coli* genomes in the frequency of *sen*B. TieB is an enterotoxin that was initially described in EIEC (Nataro et al., 1995). Additionally, UPEC strains carrying *sen*B have been associated with both pyelonephritis and urosepsis, suggesting that TieB may play a role in UPEC virulence in humans (Mao et al., 2012).

**Table 3:** Virulence factors encoded in DAEC, tEAEC, and commensal *E. coli* genomes.

**Virulence factor**	**DAEC**	**EAEC**	**Commensal**
	***n =* 10 (%)**	***n =* 16 (%)**	***n =* 7 (%)**
**Adhesins**			
Antigen 43	10 (100.00)^l^	16 (100.00)^§^	3 (42.86)
Calcium-binding antigen 43 homolog (Cah)	9 (90.00)^*^^l^	6 (37.5)	0 (0)
Curli	10 (100)	15 (93.75)	7 (100)
EaeH surface protein	8 (80.00)	10 (62.5)	6 (85.71)
*E. coli* common pilus (ECP)	7 (70.00)	11 (68.75)	5 (71.43)
EHEC autotransporter encoding gene A (EhaA)	0 (0)	4 (25.00)	3 (42.86)
EHEC autotransporter encoding gene B (EhaB)	9 (90.00)	16 (100)	5 (71.43)
*E. coli* YcbQ laminin-binding fimbriae (Elf/Ycb)	3 (30.00)	14 (87.50)^*^	5 (71.43)
Pyelonephritis-associated pilus (Pap)	0 (0)	3 (18.75)	0 (0)
Stg fimbriae	2 (20.00)	5 (31.25)	3 (42.86)
Toxigenic invasion loci A/ haemagglutinin from *E. coli* K1 (Tia/Hek)	3 (30.00)	4 (25.00)	0 (0)
Type 1 fimbriae	8 (80.00)	11 (68.75)	6 (85.71)
Trimeric autotransporter adhesin UpaG	4 (40.00)	5 (31.25)	6 (85.71)^§^
Autotransporter adhesin UpaH	1 (10.00)	0 (0)	0 (0)
**Bacteriocins**			
CcdA/CcdB type-II toxin-antitoxin system	8 (80.00)	14 (87.50)	4 (57.14)
Microcin H47 (MccH47)	0 (0)	7 (43.75)^*^	0 (0)
**Complement resistance/Immune system evasion factors**			
Increased serum survival protein (Iss)	8 (80.00)	7 (43.75)	3 (42.86)
Group II Capsule (KPSMII)	8 (80.00)	6 (37.5)	2 (28.57)
Outer membrane protein A (OmpA)	10 (100)	16 (100)	7 (100)
Protein involved in colonization (Pic)	0 (0)	13 (81.25)^*^^§^	0 (0)
TIR domain-containing protein C (TcpC)	1 (10.00)	0 (0)	0 (0)
Plasmid-encoded outer membrane protein TraT	9 (90.00)^*^	6 (37.5)	3 (42.86)
**Iron acquisition factors**			
Aerobactin	8 (80.00)	10 (62.5)	2 (28.57)
*E. coli* hemin uptake system (Chu)	6 (60.00)^*^	3 (18.75)	2 (28.57)
Enterobactin	10 (100)	16 (100)	7 (100)
Hemoglobin-binding protease (Hbp)	1 (10.00)	0 (0)	0 (0)
Sit iron/manganese transport system	8 (80.00)^*^	5 (31.25)	3 (42.86)
Yersiniabactin	10 (100)^l^	15 (93.75)^§^	2 (28.57)
**Secretion systems**			
Aai Type VI secretion system	0 (0)	14 (87.50)^*^^§^	0 (0)
*E. coli* Type III secretion system 2 (ETT2)	4 (40.00)	2 (12.50)	0 (0)
**Toxins**			
EAEC heat-stable enterotoxin 1 (EAST1)	1 (10.00)	9 (56.25)^*^^§^	0 (0)
α-Hemolysin (HlyA)	0 (0)	4 (25.00)	0 (0)
Hemolysin E (HlyE)	6 (60.00)	13 (81.25)^§^	1 (14.29)
Plasmid-encoded toxin (Pet)	0 (0)	3 (18.75)	0 (0)
Secreted autotransporter toxin (Sat)	6 (60.00)^l^	8 (50.00)	0 (0)
Secreted serine protease A (SepA)	0 (0)	3 (18.75)	0 (0)
*Shigella* enterotoxin 1 (ShET1)	0 (0)	13 (81.25)^*^^§^	0 (0)
Shigella IgA-like protease homolog (SigA)	0 (0)	2 (12.50)	1 (14.29)
Enterotoxin TieB	4 (40.00)^*^	0 (0)	2 (28.57)
Vacuolating autotransporter toxin (Vat)	1 (10.00)	0 (0)	0 (0)
**Other**			
*Escherichia coli* putative arylsulfatase (AslA)	1 (10.00)	13 (81.25)	3 (42.86)^l^
Dispersin	0 (0)	15 (93.75)^*^^§^	0 (0)
Dispesin translocator (Aat)	0 (0)	15 (93.75)^*^^§^	0 (0)
ETEC autotransporter A (EatA)	1 (10.00)	0 (0)	0 (0)
*Shigella flexneri* Homolog Shf	1 (10.00)	7 (43.75)	2 (28.57)

* *P < 0.05 between DAEC and EAEC, Fisher exact test*.

l *P < 0.05 between DAEC and commensal, Fisher exact test*.

§ *P < 0.05 between EAEC and commensal, Fisher exact test*.

Similarly, the gene encoding for the plasmid-encoded outer membrane protein TraT was more prevalent in DAEC than in tEAEC (90 vs. 37.5%, *P* = 0.0143). TraT is a complement-resistance molecule that is believed to enhance *E. coli* serum resistance by inhibiting later stages of the membrane attack complex activity or by altering C3 deposition on the bacterial surface and affecting outer membrane permeability (Miajlovic and Smith, 2014). *tra*T was detected in a significantly higher proportion of *E. coli* strains isolated from patients with bacteraemia/septicaemia and enteric infections when compared to *E. coli* strains isolated from healthy subjects (Montenegro et al., 1985).

IAS is the most prevalent functional group associated with DAEC and we identified two genes that were significantly more prevalent in DAEC than in tEAEC genomes: Chu, which binds host hemoproteins (60 vs. 18.8%, *p* = 0.0461), and Sit iron/manganese transport system, for the utilization of ferric iron (80 vs. 31.25%, *p* = 0.0414) (Table 3). Mice infected with *chu*A-inactivated UPEC strains resulted in attenuation of UPEC virulence *in vivo*, indicating that this heme receptor is a VF in UPEC (Torres et al., 2001). Sit has been described as the sole iron-uptake system in *S. flexneri* that allows this strain to survive and form plaques in a monolayer of eukaryotic cells, suggesting a direct role in *S. flexneri* pathogenesis (Runyen-Janecky et al., 2003). Furthermore, in *S. enterica* serovar Typhimurium the Sit system transports manganese (Kehres et al., 2002).

In contrast with DAEC, toxins play an important role in tEAEC virulence (Estrada-Garcia and Navarro-Garcia, 2012). As shown in Table 3, ShET1 (81%) and HlyA (25%) were only identified among tEAEC genomes, as both toxins play a role in tEAEC pathogenesis. ShET1 is an oligomeric toxin that induces intestinal secretion via intracellular increase of cAMP and cGMP (Fasano et al., 1997). Sat toxin, previously reported to be significantly associated with DAEC strains (Meza-Segura and Estrada-Garcia, 2016), was similarly distributed among the genomes of both pathotypes (Table 3).

The Protein involved in colonization (Pic) was exclusively found and highly prevalent (81%) in tEAEC. Pic and ShET1 are encoded on the same chromosomal locus, encoded on opposite strands. Pic was first described in tEAEC (Henderson et al., 1999), since then it has been identified in EHEC, EPEC, and UPEC, as well as in other pathogens of the Enterobacteriaceae family (Abreu and Barbosa, 2017). Pic is a secreted autotransporter able to cleave C2, C3/C3b, and C4/C4b, and works synergistically with human Factor I and Factor H (FH), thereby promoting inactivation of C3b. It is thereby considered a CR/ISEF (Abreu and Barbosa, 2017). Also, *E. coli* Ycb laminin-binding fimbriae (Elf/Ycb) was significantly more frequent among tEAEC than DAEC genomes (87.5 vs. 30%, *P* = 0085). Elf contributes to the adherence of STEC to human intestinal epithelial cells, and to cow and pig gut tissue *in vitro* (Samadder et al., 2009). To the best of our knowledge, this is the first time Elf/Ycb has been described among DAEC and tEAEC strains. As expected, the genes encoding for dispersin (*aap*) and its translocator (*aat*A) were significantly associated with tEAEC (Patzi-Vargas et al., 2015). Obviously, the presence of these virulence genes should be corroborated among tEAEC and DAEC strains from other regions of the world, to confirm its importance in the pathogenesis of these strains.

### Association of DAEC and tEAEC Virulence Genes With Clinical Severity of Diarrheal Disease

The presence of 29 *E. coli* virulence genes, identified by PCR, among the 38 DAEC and 30 tEAEC strains is shown in Table 4. In agreement with the comparative genome analysis, genes encoding for ChuA and SitA were more prevalent among DAEC isolates, while AaiC (T6SS), AatA, dispersin (*aap*), EAST1 (*ast*A), MccH47 (*mch*B) and Pic, among tEAEC isolates. In addition, PCR results revealed for the first time the presence of five genes significantly associated with DAEC strains: *iss, kps* MII, *fyu*A, and *iut*A. On the other hand, *hly*A, *pap*C, *pet* and *sep*A were significantly associated with tEAEC isolates.

**Table 4:** Prevalence of 29 selected virulence genes among DAEC and tEAEC strains identified as the only pathogens isolated from children with diarrhea.

**Group**	**Gene**	**DAEC, *n =* 38 (%)**	**tEAEC, *n =* 30 (%)**
**Adhesins**	*agn*43	36 (94.74)	30 (100)
	*fim*A	27 (71.05)	23 (76.67)
	*pap*C	1 (2.63)	6 (20.00)^*^
**Bacteriocins**	*ccd*B	26 (68.42)	26 (86.67)
	*mch*B	0 (0)	9 (30.00)^*^
**Complement resistance/Immune system evasion**	*iss*	25 (65.79)^*^	12 (40.00)
	*kps*MII	28 (73.68)^*^	13 (43.33)
	*pic*	0 (0)	21 (70.00)^§^
	*tra*T	23 (60.53)	17 (56.67)
**Dispersin**	*aap*	2 (5.26)	29 (96.67)^§^
**Dispesin translocator**	*aat*A	0 (0)	28 (93.33)^§^
**Iron acquisition systems**	*iro*N	0 (0)	0 (0)
	*chu*A	22 (57.89)^*^	8 (26.67)
	*fyu*A	38 (100)^*^	24 (80.00)
	*iut*A	33 (86.84)^*^	13 (43.33)
	*sit*A	24 (66.16)^§^	3 (10.00)
**Secretion systems**	*aai*C	0 (0)	21 (70.00)^§^
	*eiv*A	12 (31.58)	6 (20.00)
**Toxins**	*ast*A	4 (10.53)	13 (43.33)^*^
	*cdt*B	0 (0)	0 (0)
	*cnf*1	0 (0)	1 (3.33)
	*hly*A	0 (0)	8 (26.67)^*^
	*hly*E	18 (47.37)	18 (60.00)
	*pet*	0 (0)	5 (16.67)^*^
	*sat*	23 (60.53)	11 (36.67)
	*sep*A	0 (0)	4 (13.33)^*^
	*sig*A	0 (0)	2 (6.67)
	*sub*AB	0 (0)	0 (0)
	*vat*	2 (5.26)	0 (0)

* *P < 0.05, Fisher exact test*.

**#x000A7;:** *P < 0.0001, Fisher exact test*.

*agn43, Antigen 43; fimA, Type I fimbriae; papC, P fimbriae; ccdB, CcdA/CcdB type-II toxin-antitoxin system; mchB, Microcin H47; iss, Increased serum survival protein; kpsMII, group II Capsule; pic, Protein involved in colonization; iroN, Salmochelin; chuA, E. coli hemin uptake system; fyuA, Yersiniabactin; iutA, Aerobactin; sitA, Sit iron/manganese transport system; aaiC, Aai-type VI secretion system subunit C; eivA, E. coli Type III secretion system 2 subunit eivA; astA, EAEC heat stable toxin 1; cnf-1, Cytotoxic Necrotizing Factor-1; cdtB, Cytolethal Distending Toxin; hlyA, α-Hemolysin; hlyE, Hemolysin E; pet, Plasmid encoded toxin; sat, Secreted autotransporter toxin; sepA, Secreted serine srotease A; sigA, Shigella IgA-like protease homolog; subAB, Subtilase cytotoxin; vat, Vacuolating autotransporter tox*.

**Figure 3:**
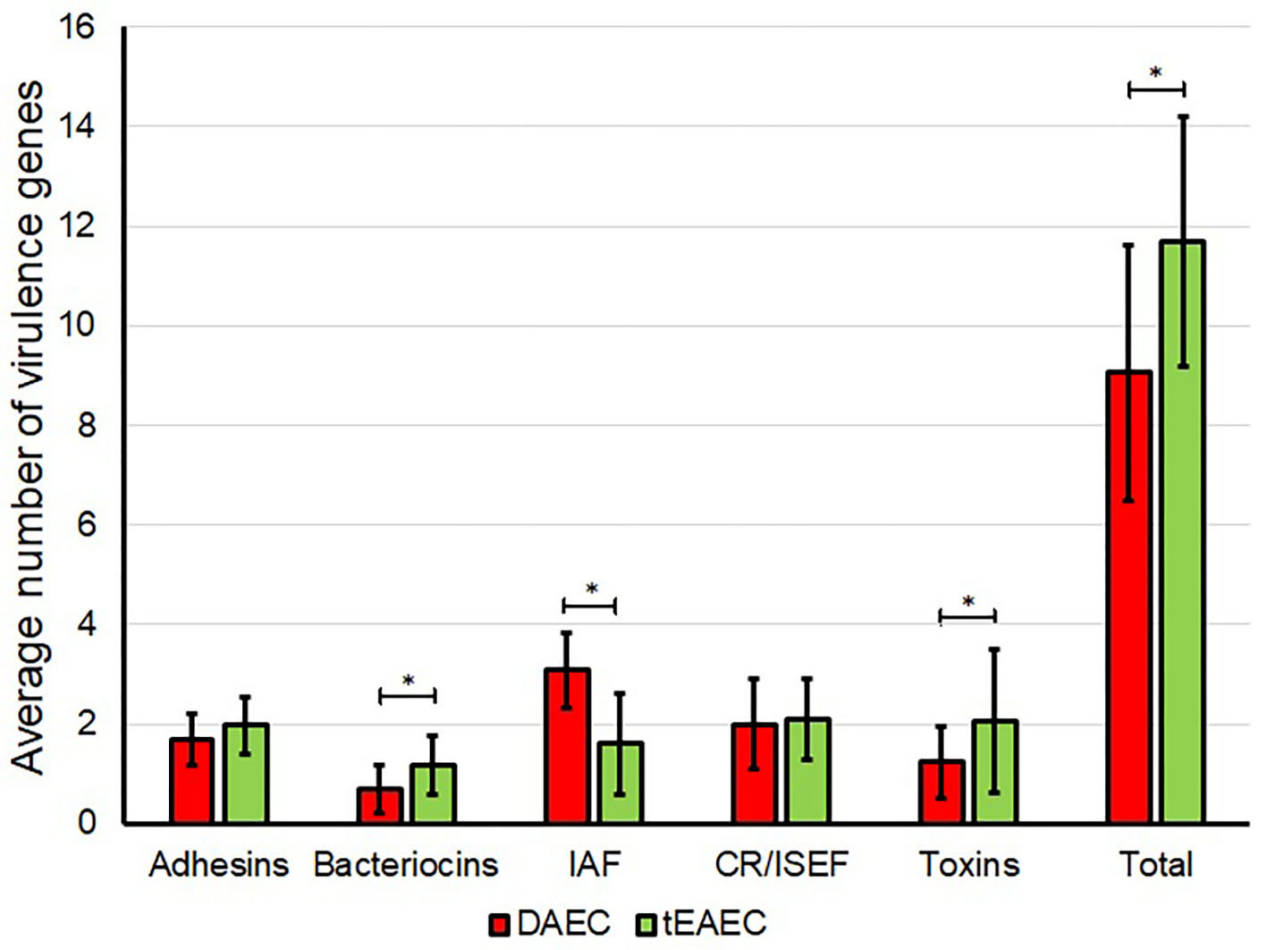
Distribution of virulence genes in DAEC and tEAEC strains. The average number of different groups of virulence genes detected by PCR in DAEC (red) and tEAEC (green) strains are shown. Asterisks indicate a significant difference in the number of virulence genes between both pathotypes (*P* < 0.05, MWUT). CR/ISEF, Complement resistance/Immune system evasion factors; IAF, Iron acquisition factors.

DAEC strains harbored significantly more IAS encoding genes, while tEAEC strains harbored more toxin and bacteriocin genes (Figure 3), as previously shown in the ORF genome analysis (Table 2).

Of the 38 DAEC strains, 20 (52.6%) had the *afa*C*-ang*43*-fyu*A*-iut*A*-kps*MII*-sat-fim*A virulence gene profile (VGP), which harbors two genes encoding for IAS yersianiabactin (*fyu*A) and aerobactin (*iut*A) siderophore receptors. Both yersiniabactin and aerobactin genes are found more frequently among pathogenic *E. coli* strains and have been involved in UPEC virulence (Garcia et al., 2011; Flores-Mireles et al., 2015). Yersiniabactin binds copper to protect pathogens during infection; direct mass spectrometroscopy showed that it also binds nickel, cobalt, and chromium, and transports these metals by its receptor FyuA (Koh et al., 2017). Detection of urinary Ybt and serological detection of the outer membrane Ybt importer (*fyu*A) have been implicated in clinical UTI (Koh et al., 2017). It also appears that the aerobactin system is important in UPEC virulence (Gao et al., 2012). Therefore, the presence of several IAF in DAEC genomes should be advantageous not only for its virulence, but to overcome the host “nutritional immunity” which limits bacterial growth by sequestering metals in the intestine (Lopez and Skaar, 2018). *sat, kps*MII, and *fim*A, also included in this common VGP, belong to the functional groups of toxins, CR/ISEF and adhesins, respectively. In animal models, SAT-producing UPEC and DAEC strains may cause damage to the renal and intestinal epithelium, respectively (Guyer et al., 2002; Taddei et al., 2005). Seventy percent of NMEC strains harbored the *kps*MII gene that encodes for the K2 capsule (Wijetunge et al., 2015). K2 provides protection against complement-mediated killing of UPEC CFT073 strain and has been shown to be important for UTI pathogenesis (Buckles et al., 2009). Among UPEC strains it has been shown that type 1 pilus (*fim*A) participates in the colonization of both the uroepithelium and the intestinal epithelium (Flores-Mireles et al., 2015; Spaulding et al., 2017). Furthermore, the type 1 pilus plays an essential role in ETEC virulence, acting in concert with specific ETEC colonization factors (CF) promoting optimal bacterial adhesion to cultured intestinal epithelium and to epithelial monolayers differentiated from human small intestinal stem cells (Sheikh et al., 2017).

Of the 30 tEAEC strains, *agg*R-*ang*43-*aap*-*aat*A-*aai*C-*pic*-*ccd*B was the predominant VGP (56.67%). *pic* also comprised part of the most prevalent gene profile (*set1*A-*set1*B-*pic*) identified in tEAEC strains isolated from Peruvian children with diarrhea, but not in strains isolated from asymptomatic children, and was also significantly associated with both acute diarrhea and prolonged diarrhea (Duan and Mukherjee, 2016). Pic's role in EAEC pathogenesis is multi-factorial: it is a mucinase, induces intestinal mucus hypersecretion, and is involved in gut colonization and complement inactivation; thus it appears that *pic* is a potential marker for virulent tEAEC strains (Estrada-Garcia and Navarro-Garcia, 2012; Abreu and Barbosa, 2017). CcdA/CcdB toxin-antitoxin system may increase the probability of tEAEC colonization by inhibiting the growth of competing organisms in the gut. To the best of our knowledge, this is the first study to identify this bacteriocin among tEAEC strains.

### Specific DAEC or tEAEC VGP and Adhesins Were Associated With Fever, Age, Hyponatremia, and Disease Severity

We explored associations between the clinical characteristics of the diarrheal episode and specific VGP in DAEC and tEAEC. The *agn*43-*ccd*B-*fyu*A-*iut*A-*kps*MII-*sat*-*sit*A-*tra*T DAEC VGP was significantly associated with fever ≥ 38°C (7/12, 58.3% vs. 3/26, 11.5% *p* = 0.0047). It is unknown whether any of the molecules encoded by these genes directly induces secretion of IL-1, the cytokine that induces fever (Endres et al., 1987). However, we have previously shown that both DAEC reference strain C1845 and a clinical DAEC isolate induce the production of IL-1ß from Caco-2 confluent cells *in vitro* (Patzi-Vargas et al., 2013). Furthermore, DAEC and tEAEC strains isolated from patients with fever had a higher number of toxin-encoding genes (RF 2.0 vs. 1.3, *P* = 0.0231) and overall more virulence genes (RF 11.5 vs. 9.43, *P* = 0.0037) than strains isolated from children without fever.

The *aap*-*aat*A-*agn*43-*fim*A-*fyu*A VGP was only found among tEAEC isolates from children aged < 2-years (18/24, 75%, *P* = 0.0157), while the *mch*B-*hly*E-*iut*A-*kps*MII VGP was exclusive to older children (3/5, 60%, *P* = 0.0027). It has been suggested that DAEC strains from children and adults constitute two different populations (Mansan-Almeida et al., 2013); this may be the case for tEAEC strains as well. *Sat* was found to be associated with hyponatremia in tEAEC diarrhea (4/5, 80% vs. 7/25, 28%, *p* = 0.0472). In a ligated rabbit ileal loop assay, SAT produced a copious amount of fluid similar to that observed with ETEC LT toxin, as well as villous edema, vacuolization and loss of internal villous structure (Taddei et al., 2005), that may result in loss of electrolytes and hyponatremia. In both DAEC and tEAEC strains, *fim*A (type 1 pilus) was more common in isolates from children ≤ 24 months than in older children (22/27, 81.5% vs. 5/11, 45.45%, *P* = 0.0471; and 22/24, 91.67% vs. 1/6,16.67%, *P* = 0.0008; respectively). As observed for ETEC, type 1 pilus may enable more effective bacterial adhesion of DAEC and tEAEC strains to the intestinal epithelium of younger children (Sheikh et al., 2017). tEAEC strains isolated from cases with dehydration signs had a higher number of genes encoding for adhesins (RF 3.43 vs. RF 2.74, *P* = 0.0126).

### Hypothetical DAEC Model of Pathogenesis

**Figure 4:**
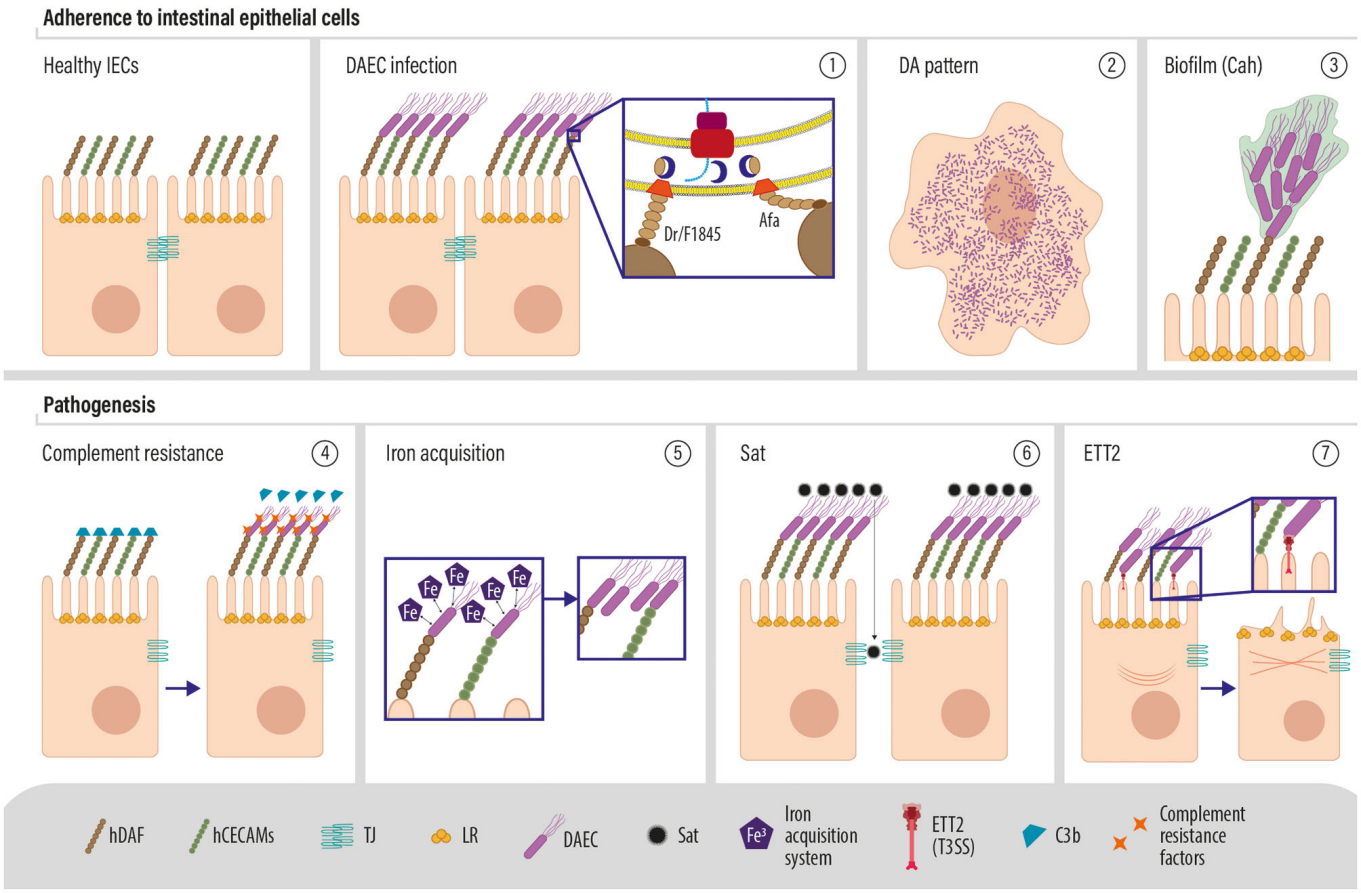
Molecular pathogenesis of DAEC (1). DAEC expresses Afa/F1845/Dr adhesins that interact with the decay accelerating factor (also known as Dr or CD55) and (2) are responsible for the characteristic diffuse adherence (DA) pattern exhibited by this pathotype (3). The production of Cah may assist in the initial adherence to enterocytes or with production of biofilm (3). Adherence of the bacteria may dampen the inhibitory activity of hDAF, which could result in a decreased inactivation of the complement. However, DAEC strains carry a wide variety of complement resistance factors, which might protect the bacteria (4). Iron acquisition systems are common among DAEC, suggesting an important role for iron scavenging in DAEC infection, which may facilitate bacterial replication and colonization (5). The serine protease Sat is also common among DAEC strains and it is associated with the loss of cell shape and potentially detachment from epithelial layers. Finally, the ETT2 type 3 secretion system may deliver intracellular effectors to the enterocytes, which may induce cytoskeletal modifications.

Based on the variety of novel genes identified among DAEC genomes, we propose the following model for DAEC pathogenesis (Figure 4). Afa/F1845/Dr adhesins bind to hDAF/CD55 and hCEACAMs, localized on the apical pole of human intestinal epithelial cells, which results in DAEC characteristic diffuse adherence pattern. A novel adhesin, Cah, was found among most DAEC strains, suggesting that it could also participate in the initial adherence of DAEC, in the aggregation of bacteria and in the formation of biofilm, as it has been described before for STEC (Torres et al., 2002; Carter et al., 2018). Also, Cah shares high sequence similarity with AIDA-1, an adhesin that also mediates DAEC diffuse adherence pattern to HeLa cells (Torres et al., 2002). DAEC intrinsically has an effect on the host complement system since it binds hDAF/C55, hampering the capacity of this molecule to inhibit the complement-cascade amplification. The expression of CR/ISEF by DAEC could protect the bacteria from the action of the complement. For example, *tra*T, which inhibits later stages of the membrane attack complex activity, was highly prevalent among DAEC strains. Moreover, these strains also express a wide variety of iron acquisition systems, such as Chu, which binds host hemoproteins, and Sit iron/manganese transport system for the utilization of ferric iron, allowing DAEC to capture these elements that are indispensable for bacterial growth and to replicate faster than resident microbiota. Also, the most common DAEC VGP *afaC-ang43-fyuA-iutA-kpsMII-sat-fimA*, contained two IAS, the yersianiabactin and aerobactin siderophore receptors.

Among DAEC strains, we found the whole gene cluster encoding ETT2, which could be potentially involved in the polymerization of actin observed during DAEC infection (Riveros et al., 2011), as it has been shown for the type three secretion system of EPEC and EHEC (Kaper et al., 2004). Sat, the only previously described DAEC VF, was also identified in the most common DAEC VGP. Sat may be responsible for tight junction alteration and increased secretion of fluids into the lumen during the course of DAEC infection. We identified a gene encoding for a second toxin, TieB, for which a mechanism of action has not yet been described.

## Conclusions

Comprehensive WGS and identification of VF for DAEC and tEAEC strains reveal for the first time that these two pathotypes are phylogenetically related. Given this relationship, we hypothesized that the two pathotypes would harbor a shared set of VF (as do EPEC and STEC, for example), supplemented by different factors that mediate their divergent pathogenic lifestyles. This was in fact the case, as most VF were shared between the two pathogens with few exceptions. Moreover, our work is the first comprehensive genomic analysis of DAEC, and as such identified several novel VF groups and genes among DAEC strains, including the ones encoding for Cah, Iss, Kps MII, Sit, TieB, and TraT; the analysis has allowed us to suggest for the first time a pathogenetic paradigm for this pathotype. Lastly, our work suggests clinical application by identifying common and conserved VF that may offer promise as immunogens to control DAEC and tEAEC infections.

## Data Availability Statement

The genome sequence assemblies generated in this study have been deposited in GenBank under the accession numbers listed in Supplementary Table 2. The remaining data that support the findings of this study are available from the corresponding author upon request.

## Ethics Statement

The studies involving human participants were reviewed and approved by both the Hospital General O'Horan Ethics Committee and the CINVESTAV Committee of Bioethics for Human Research. Legal guardians were required to sign an informed consent form. All children received medical treatment according to the hospital protocols. Written informed consent to participate in this study was provided by the participants' legal guardian/next of kin.

## Author Contributions

MM-S and TE-G conceived the experiments. TE-G was Co-PI of the epidemiological study. MM-S and NM-G performed the experiments. MM-S, AV-P, and EM-R performed all bioinformatic analysis. MBZ was Co-PI of the epidemiological study, collected clinical samples data, and strains data. MM-S, MBZ, JPN, and TE-G wrote the manuscript, which was reviewed and approved by all authors. All authors contributed to the article and approved the submitted version.

## Conflict of Interest

The authors declare that the research was conducted in the absence of any commercial or financial relationships that could be construed as a potential conflict of interest.

## Footnotes

^1^Krueger F. *Trim Galore 0.4.1*. Available online at: https://www.bioinformatics.babraham.ac.uk/projects/trim_galore/ (accessed on November 12th, 2015).

^2^Available online at: https://github.com/avera1988/E.coli_PopulationStructure.
