# Supplementary material for: New Insights Into DAEC and EAEC Pathogenesis and Phylogeny
Source: Front Cell Infect Microbiol. 2020 Oct 15;10:572951. doi: 10.3389/fcimb.2020.572951 (PMC7593697; doi:10.3389/fcimb.2020.572951)
Supplement: Supplementary file 2 [file Data_Sheet_1.pdf]

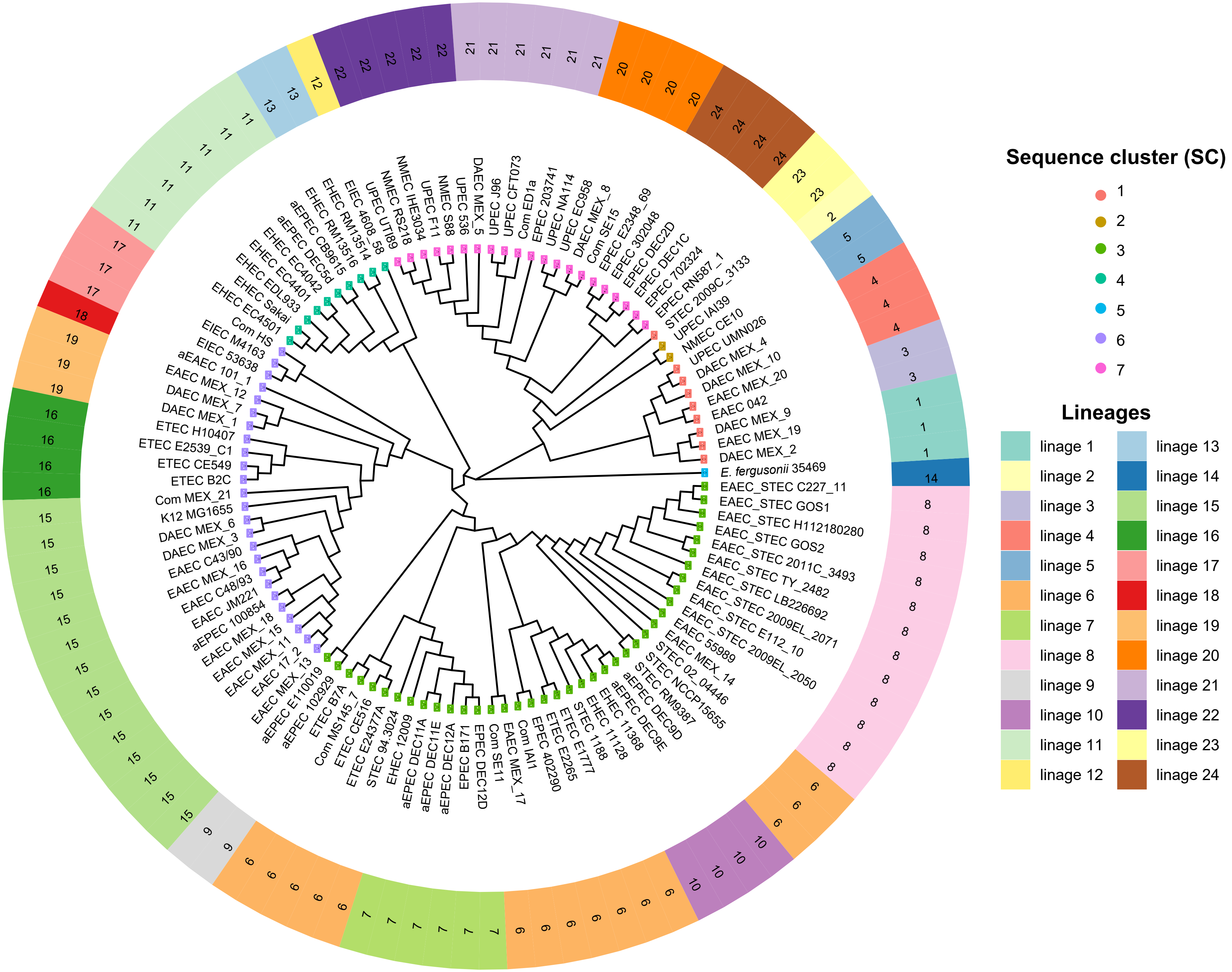

Supplementary Figure 1. Population structure of *E. coli*. A maximum likelihood tree was constructed based on 1,144 single copy genes from the *E. coli* core-genome as described in Materials and Methods. *E. fergusonii* 35469 was used as an out-group. Population structure was calculated using a Bayesian approach by RhierBAPS package (Tonkin-Hill et al., 2018) in R 3.6.3. Seven different sequence clusters (SC) were identified, which were further divided into 24 lineages (outer colored ring). Numbers on the outer colored ring indicate the corresponding lineage. All scripts to replicate this experiment are deposited in Github (https://github.com/avera1988/E.coli_PopulationStructure).
